# Breastfeeding and child survival from 0 to 5 years in Côte d'Ivoire

**DOI:** 10.1186/s41043-020-0210-4

**Published:** 2020-03-30

**Authors:** Yomin Virginie Yapo

**Affiliations:** Department of Economics and Management, Félix Houphouët Boigny University, 01 BP V43 Abidjan 01, Abidjan-Cocody, Côte d’Ivoire

**Keywords:** Exclusive Breastfeeding, Child survival, Health, up to 6 months, Mother, Côte d’Ivoire

## Abstract

**Background:**

One of the main objectives of health policy-makers is to promote children’s growth, development, and survival. The current research evaluates the impact of breastfeeding on infant survival and highlights the major socio-economic determinants of child survival from 0 to 5 years old in Côte d’Ivoire.

**Methods:**

This study uses Probit estimation to evaluate the impact of the type of breastfeeding on the survival of children aged from 0 to 5 years old. The main socio-economic determinants of child survival were identified and analyzed. The sample of the study covers 7776 children under 5 years old drawn from the Côte d'Ivoire Demographic Health Surveys and the Multiple Indicators cluster survey of 2012.

**Results:**

A child is more likely to survive when immediate exclusive breastfeeding was practiced for up to 6 months. The probability of survival increases significantly when the mother lives in a healthy environment, when she has at least a primary school education, and when she plays a leading role in caring for the children. Likewise, when she better controls the market of some breast milk supplement and she chooses the best milk formula to complete feeding for her baby, the child’s chances of survival increase significantly.

**Conclusion:**

Health policy-makers must strengthen programs to promote exclusive breastfeeding up to 6 months through social campaigns. It should also strengthen the capacity of health workers (midwives, nurses, doctors, etc.) to better guide and provide training to mothers and young women about childbearing age to allow them to practice exclusive breastfeeding for up to 6 months. It is only after 6 months that they have to complete infant feeding by providing some semi-solid food rich in vitamins, proteins, and minerals. Taking into account the time constraint when they are engaged in economic activity, they must choose the best formula milk to supplement breastfeeding. It is also important to educate women to improve hygiene in their housing, in their neighborhood and in their community in order to promote the welfare and health of their children.

## Background

Health is one of the priority areas of sustainable human development. It is part of the Millennium Development Goals (MDGs). Indeed, MDG 1 and MDG 4 focus on reducing malnutrition and infant mortality. Thus, most of the development stakeholders are aware of this real fact and attempt to address health issues worldwide. In developed countries, health indicators display quite a satisfactory rate that resulting from concrete actions that have been carried out for the population regarding children and mothers. In these countries, the mortality of children under 5 years old has declined by half. In other words, the number of child deaths decreased from 90 to 46 per 1000 live births in 2013 according to the estimates [1]. However, in developing countries, particularly in Sub-Saharan Africa, the mortality rate of children under five is high [1]. In Sub-Saharan Africa, the probability of children to die before they turn 5 is 14 times higher than in developed countries [2]. Sub-Saharan Africa has the highest child mortality rate in the world with 1 child out of 12 who dies before his fifth birthday [3]. In Côte d’Ivoire, the rate of mortality under 5 years old varies respectively from 106 to 103, 99, 96, and to 93 per thousand live births in 2011, 2012, 2013, 2014, and in 2015 [4]. Regarding infant mortality, the rates vary from 104.9 to 99.5, 84.1, 81.3, 79.0, 76.9, 75.0, 72.8, 70.6, 68.5 and to 66.6 (per 1000 live births), respectively, in 1990, 2000, 2007, 2008, 2009, 2010, 2012, 2014, and in 2015 [3]. The downward trend in mortality is the positive result of the measures taken by the government on public health. During the same period, progress has been made in the health sector in Côte d’Ivoire, particularly in Mother and Child Protection Services, where access to the health care system is free. Health care is supported by public hospitals^1^. Cesarean delivery is free in public health centers and the cost of normal birth is reduced [6].

Furthermore, to promote children’s growth, development, and survival, WHO and UNICEF recommend exclusive breastfeeding during the first 6 months after birth. In the implementation of this recommendation, since 1991, the Ivorian public health authorities have reinforced the breastfeeding program with the initiative “Hospitals Babies’ Friends” [7]. This is a global strategy, developed jointly by WHO and UNICEF whose goal is to protect, encourage, and promote breastfeeding. In Côte d’Ivoire, many hospitals have supported breastfeeding through this initiative, thereby increasing the number of breastfeeding mothers [8]. In the same context, since 1995, the World Breastfeeding Week is celebrated every year among women of childbearing age, especially mothers on practicing exclusive breastfeeding up to the first 6 months of child’s life [9]. The Ivorian government continues to intensify through campaigns, lectures, debates, workshops and seminars, and promotion of exclusive breastfeeding across the country [8]. Exclusive breastfeeding is a decline factor in infant mortality [10]. In fact, breast milk is the first safe food for children aged between 0 and 2 years old [11]. It covers all the nutritional requirements of infants while protecting them against infectious diseases [11]. Breastfeeding promotes children’s sensory and cognitive development [12].

Several studies show that breastfeeding is a child mortality reduction factor. Filmer and Pritchett [13] who say that drinking water and quality infrastructure are factors to improve children’s health under 5 years old. Economic development implies access to basic social services such as schools, health facilities, access to drinking water, and road infrastructure. Thus, Christopher Grigoriou [14] shows the positive impact of economic development on child mortality. Despite the growth in the 1990s in Burkina Faso, Lachaud [15] argues that poverty still persisted. He conducted a study to explain the link between changes in child survival and the persistence of poverty. In this study, he emphasizes the determinants of infant and child survival. They are the standard of living of households in terms of assets, education of the mother, the geographical location of households, childbirth assisted by qualified medical staff, mothers’ age during birth, intergenesic interval, multiple births, and birth rank. Among these factors, the mother’s education draws the attention of most authors who worked on these issues. They focus on the role of educated mothers on increasing child survival chances. In his study in Bobo Dioulasso (Burkina Faso), Banza Baya [16] shows that parental education significantly increases the chances of child survival. The mother’s education acts through several channels. Economically and socially, formal instruction enables households to obtain gainful employment, secured source of the welfare of the family, children in particular. Culturally, formal instruction promotes access to information by the media concerning practical measures to improve the nutritional status and health of children.

Furthermore, in addition to socio-economic determinants of child survival, some authors emphasize the feeding modes that can significantly contribute to maintaining the child’s good health. In this perspective, regarding children with ages between 0 and 1 year old, health officials consider breastfeeding as the first and best infant food [11]. In this conception, after a sociological analysis, Bayard [12] emphasizes that breast milk provides benefits for mother and child on the physical, psychological, emotional, and cognitive aspects. Breast milk completely covers the nutritional needs of infants and is one of the best investments regarding child survival, since its cost lies primarily in the mother’s diet [17]. Breast milk is known for its nutritional value (vitamins, minerals, proteins, fats, carbohydrates) and immune [18]. Breastfeeding and birth spacing have significant effects on child survival in China [19]. The act of birth planning allows the mother to increase the duration of breastfeeding at the first child level under the age of 2 years old and over according to the recommendation of the World Health Organization [1]. The practice of breastfeeding fight against intestinal and respiratory infectious diseases [11]. Breastfeeding is associated with a reduced risk of gastrointestinal infections [11]. The protective effect is higher against gastrointestinal and intestinal infections than against respiratory infections and increases with exclusive breastfeeding [11]. Breast milk is a complete food necessary for the child’s growth, development, and survival [5]. Breastfeeding is the most effective way to save the life of infants [10]. It is also a means of preventing early malnutrition compared to artificial milk [11]. It is therefore the best mortality reduction factor. Breastfeeding in the early hours preceding the birth and exclusive breastfeeding for the first 6 months are key interventions to achieve MDG 1 and MDG 4. These authors continue their analysis and show that breastfeeding produces long-term effects. According to them, this mode of feeding plays a preventive role regarding certain diseases that may occur during adulthood, such as hypertension, cardiovascular disease, and obesity.

Most authors emphasize the importance of breastfeeding during the first 6 months of infant life. However, in the economic literature, few empirical studies highlight the positive impact of healthy environment on child survival. Our contribution is to show that a healthy environment is also one of the key factors in child survival after the crucial role that exclusive breastfeeding plays in the child’s growth process.

Despite the government's efforts in urban sanitation, the mentality and the behavior of the Ivorian people do not change. In the neighborhoods as well as inside the city, several people throw rubbish at the roadside. Waste management is poorly designed in Côte d’Ivoire. Much work remains to be done. An unhealthy environment is the source of several diseases such as typhoid fever, cholera, dysentery, and measles. These are diseases created by microbes (bacteria, viruses). Children aged 0 to 5 years old are very vulnerable compared to poor living environment. So, our study places particular emphasis on showing the impact of a healthy environment on the survival of children specifically.

In general terms, using an econometric approach our study evaluates the impact of breastfeeding on infant survival and highlights some major socio-economic determinants of child survival from 0 to 5 years old.

The interest of this analysis lies in the choice of the variables of the estimation model. This will allow us to answer our questions. Hence, we are in need of a methodology for analysis.

## Methods

In this section, the data, the Probit model and the specification of variables will be presented.

### Data

The third Demographic and Health Survey in Côte d’Ivoire (DHS-CI-III) combined with the Survey by Multiple Indicator Cluster Survey (MICS) was conducted from December 2011 to May 2012 across the country. In this survey, 10,060 women aged from 15 to 49 years old were successfully interviewed and so were about 5135 men aged from 15 to 59 years old. The interview consists of asking some questions to women in order to get information concerning their children’s health. Eligible children are those whose age is between 0 and 5 years old, the children who have not reached their fifth birthday (children age 0–4). Here, ages are expressed in completed years, whether the age of women, men or children. In total, 7776 children are involved. The surveys consist of filling three types of questionnaires- questionnaire for the household, for the women and for the children. The women’s questionnaire and that of the child depend on the household questionnaire. All the people who were surveyed in a household are related to the chief of the household. The chief of the household can be a man or a woman. The interviews were undertaken following a cluster sampling from the study population. A cluster consists of 25 households, which is to say in a cluster, 25 households are surveyed. These surveys were conducted by the National Institute of Statistics (INS) with technical assistance from ORC Macro, which is responsible for the international program of DHS. There are therefore national survey data. These are secondary data available in the archives of the DHS.

The DHS-MICS provides information on several areas such as sexual health, fertility preferences, knowledge, and use of family planning methods. During this survey, data were collected on breastfeeding practices, nutritional status of women and children under 5 years old, infant mortality, maternal mortality, and the health of the mother and the child. The survey also provides information on knowledge, attitudes, behavior on HIV/AIDS, other sexually transmitted diseases (STDs), and the use of mosquito nets against malaria. HIV, anemia, and malaria tests were also done during the survey.

### Probit model and specification of variables

From agricultural household surveys, Singh and Strauss [20]; Pitt and Rosenzweig [21] seek to assess food prices change effects and health actions on the health status or nutritional status of household members including children. In the case of children’s health, the factors of production or inputs used to produce their health are as following, food, access to curative and preventive care, access to drinking water, healthy environment, etc. [22]. We are inspired by the theoretical model of these authors and we indicate *H* as an individual’s health as follows^2^:

$$ H=f\left({P}_z,d,A,T,{U}_h,{V}_h,Y\right) $$

Where *P*_z_ is the price of health production inputs such as food, drinking water, and sanitation; *d* represents the observable characteristics of the individual, such as age, and sex; *A* represents assets or property owned by the household such as refrigerator, television set, and radio; *T* is time spent to improve one’s health status, for example, the time taken to practice sports. That is to say, an investment to be healthy; *U*_h_ represents the inherent characteristics of the community (health centers, etc.); *V*_h_ is unobservable household characteristics and *Y* is the income transfers.

### Probit model

This section presents the Probit models to be used. Let *Se* denote the dependent variable which takes on the value 1 if the child is alive and 0 in the case of death, that is

1 $$ S{e}_i=\Big\{{\displaystyle \begin{array}{c}1\kern0.48em \mathrm{if}\kern0.24em S{e}_i^{\ast }>\gamma \\ {}0\kern0.48em \mathrm{if}\kern0.24em S{e}_i^{\ast}\le \gamma \end{array}} $$

where *i* = 1, 2, …, *n* and *γ* is a benchmark indicating a kind of “survival” level that a child’s unobserved resistant capacity *Se*^∗^ should overcome in order for him to “survive”. This latent variable *Se*^∗^, though unobservable, is related to a *K* × 1 vector of observable characteristics *x*_*i*_ for child *i*. The set of explanatory variables commonly admitted in the child health literature can be classified in different groups, including woman’s status, woman’s human capital, existence of a healthy environment, nutrition factors and other food-related factors. The variables selected in the current study are the following:

- *allait1*, exclusive breastfeeding;
- *alaitmixte*, for breastfeeding mixed with other milks;
- *Alaitem*, breastfeeding and other liquids;
- *Vitamins*, liquids or solids foods rich in vitamins;
- *Alcool*, alcohol consumption;
- *educ*, level of education (*educ1* for no schooling serving as the reference category, *educ2* for primary education and *educ3* for secondary education or more);
- *femconj*, woman living with her spouse;
- *femchef*, woman is household head;
- *habitat*, healthy environment; and
- *eausec*, drinking water.

From Equation (1), it follows that,

2 $$ S{e}_i^{\ast }={a}^{\prime }{x}_i+{\varepsilon}_i $$

with *ε*_*i*_ the associated error term and *a* a *K* × 1 vector of coefficients.

To be more specific, we can write:

3 $$ {\displaystyle \begin{array}{l}S{e}_i^{\ast }={\alpha}_0+{\alpha}_1 allait{1}_i+{\alpha}_2 alaitmixt{e}_i+{\alpha}_3 alaite{m}_i+{\alpha}_4 vitamine{s}_i+{\alpha}_5 alcoo{l}_i+{\beta}_2 educ{2}_i+\\ {}\kern3.5em +{\beta}_3 educ{3}_i+{\gamma}_1 femcon{j}_i+{\gamma}_2 femche{f}_i+\delta habita{t}_i+\eta eause{c}_i+{\varepsilon}_i\end{array}} $$

where *a* = (*α*_0_, …, *α*_5_; *β*_2_, *β*_3_; *γ*_1_, *γ*_2_; *δ*; *η*)^′^ is the vector of regression parameters to be estimated whereas *ε*_*i*_ denotes the error term for child *i*.

It is worth mentioning that the Probit model is estimated by the maximum likelihood procedure (Table 1).

**Table 1:** Specification of variables

Variables	Definition	Range value and unit	Expected sign
HS_e_	Survival probability is the dependent variable*.* Chances of child survival	Variable, in the database record B5, the value is 1 when “child is alive” 0 otherwise.	
ST_fe_ Spouse presence	Women status: it is about marital status.	Yes = 1, 0 otherwise	(+)
	Woman living with spouse: the woman’s husband is present and they live together in the household.		(+)
Household head	A woman as a household head. It is about a woman who is unmarried, widow or divorced. In the household, she is the main provider of needs.	Yes = 1, 0 otherwise	(+)
*D*_fh_ Education	Human capital endowment: level of education. The formal education levels are represented by variables—no education, primary, secondary, or higher education, which take into account the number of years schooling received by the mother at every level of the educational process.	No education = 1; primary = 2; secondary, or higher = 3	(+)
*M*_n_	Healthy environment: the living environment where the mother and child live must be healthy.	Yes = 1, 0 otherwise	
*N*_e_ Breastfeeding	Nutrition factors: immediate exclusive breastfeeding assumes that the infant only absorbs breast milk immediately the day he was born. It does not receive any other liquids or solid foods, not even water, except oral rehydration solutions, or medications.	Yes = 1, 0 otherwise	(+)
	Breastfeeding mixed with other milk: mixed breastfeeding is the association of breastfeeding with baby milk bottles. It is practiced mainly in 2 situations: either the bottle comes in complement of the breast if the mother does not have enough milk or she is busy with economic activities.	Yes = 1, 0 otherwise	(+)
	Breastfeeding and other liquids (tea, fruits juice, water, etc.). The association of breastfeeding with other liquid foods such as porridge of maize, millet and fruit juice, water, tea.	Yes = 1, 0 otherwise	(+)
Semi-liquids or semi-solids foods rich in vitamin	Vitamins: A, C, D, B_6 ,_ B_12_, and others It’s about foods rich in vitamins such as “blédina.” It is a baby food formula rich in vegetables, (carrots, green beans, etc.), rich in cereals (wheat, maize, rice, millet), and rich in milk. This also applies to African baby food rich in cereals (rice, corn, millet) and apple puree mixed with egg yolk.	Yes = 1, 0 otherwise	(+)
Alcohol	Alcohol drinks: mother gives some drinks containing alcohol to their child and when the mother puts alcohol in traditional medicine.	Yes = 1, 0 otherwise	(-)
*V*_e_ Drinking water	Others factors: drinking water is water that can be consumed without the risk of getting sick and not containing microbes (viruses, bacteria) and toxic products. Access to drinking water No access to drinking water	Yes = 1, 0 otherwise	(+)

Source: Available variables in DHS-MICS-2011-2012

## Results

First, we present the results obtained after the econometric estimations (See the Additional file 1).

The econometric results are summarized in Tables 2 and 3. At first, we highlight the relationship that exists between the probability of child survival and some types of breastfeeding. Then relevant socio-economic determinants of child survival are apprehended.

**Table 2:** Estimation of the link between probability of child survival and breastfeeding modes—Côte d’Ivoire children from 0 to 5 years. Result of Probit^1^model- STATA SE 14 software used

Independent variables	Coef	SE^2^	*P* value	95% CI	Marg eff ^3^
Constant	1.0630	0.0292	0.000	(1.0057; 1.1202)	0.9199 (0.000)
Immediate exclusive breastfeeding^4^	0.1741	0.0502	0.001	(0.0757; 0.272)	0.0243 (0.000)
Breastfeeding mixed with other milk^5^	0.4388	0.0766	0.000	(0.2886; 0.5890)	0.0682(0.000)
Breastfeeding with other liquids^6^	0.1012	0.0774	0.191	(−0.0505; 0.2530)	0.0151 (0.193)
Log likelihood	−2224.12	−	–	−	−
LR *χ*^*2*^ (11)	178.45	–	–	−	−
Prob > *χ*^*2*^	0.0000	−	–	−	−
Obs	7776	−	–	−	−

^1^In the estimation of Probit model, the dependent variable is the probability of child survival

^2^*SE* = standard error

^3^The marginal effects are partial derivatives of the characteristics—the values in parentheses are the *P* value

^4^Immediate Exclusive breastfeeding: coded 1 = yes, 0= otherwise

^5^Breastfeeding mixed with other milk: coded 1 = yes, 0 = otherwise

^6^Breastfeeding with other liquids: coded 1 = yes, 0 = otherwise

Source: From DHS-MICS-2011-2012-CI data

**Table 3:** Regression coefficients estimation and marginal effects of the determinants of child survival probability from 0 to 5 years in Côte d’Ivoire—result of Probit^1^ model- STATA SE 14 software used

Dependent variables	Coef	SE^2^	*P* value	95% CI	Marg eff ^3^
Constant	0.7352	0.0865	0.000	(0.5657; 0.9046)	0.9213 (0.000)
Immediate exclusive breastfeeding^4^	0.1663	0.0505	0.001	(0.0672; 0.2653)	0.0230 (0.000)
Breastfeeding mixed with other milk^5^	0.4645	0.0819	0.000	(0.3040; 0.6290	0.0715 (0.000)
Breastfeeding with other liquids^6^	0.0832	0.0873	0.341	(−0.0879; 0.2543)	0.01224 (0.342)
Vitamins^7^	0.0093	0.0854	0.913	(−01582; 0.1767)	0.0013 (0.913)
Alcohol^8^	0.0893	0.1285	0.487	(−0.1626; 0.3492)	0.0124 (0.460)
Level of education^9^	−	−	–	−	−
Primary	0.0809	0.0514	0.115	(−0.00197; 0.1816)	0.0115 (0.104)
Secondary and higher	0.1842	0.0803	0.022	(0.0027; 0.3416)	0.0243 (0.010)
Woman lives with spouse^10^	0.0736	0.0491	0.134	(−0.0227; 0.1699)	0.01106 (0.143)
Woman is head of household^11^	0.2344	0.1058	0.027	(0.02700; 0.44181)	0.0229 (0.009)
Healthy environment^12^	0.1373	0.0392	0.000	(0.0604; 0.2141)	0.0201 (0.000)
Drinking water^13^	–	–	--	–	–
No access	− 0.0052	0.0082	0.530	0.0213	− 0.0007
Log likelihood	− 2208.52	–	––	–	(0.0530)
LR *χ*^*2*^ (11)	209.65	–	–	–	–
Prob > *χ*^*2*^	0.0000	–	–	–	–
Obs	7776	–		–	–

^1^In the estimation of Probit model, the dependent variable is the probability of child survival

^2^SE = standard error

^3^Marginal effects are partial derivatives of the characteristics—the values in parentheses are the *P* value

^4^ Immediate exclusive breastfeeding: yes = 1, 0 = otherwise

^5^Breastfeeding mixed with other milk: yes = 1, 0 = otherwise

^6^Breastfeeding with others liquids: yes = 1, 0 = otherwise

^7^Vitamins: yes = 1, 0 = otherwise

^8^Alcohol: yes = 1, 0 = otherwise

^9^Level of education: base = no education

^10^Woman lives with spouse: yes = 1, 0 = otherwise

^11^Woman is head of household: yes = 1, 0 = otherwise

^12^Healthy environment: yes = 1, 0 = otherwise

^13^Drinking water: base = access to drinking water

Source: From DHS-MICS-2011-2012-CI data

The econometric results contained in Table 2 are from the Probit model. These results show us that a child is more likely to survive when he is exclusively breastfed immediately. The regression coefficients associated with the variable “Immediate exclusive breastfeeding” is positive and very significant (α_1_ = 0.1741, *P* < 0.01). All things being equal when the mother increases the duration of exclusive breastfeeding for a year, the child’s survival chances increase by 0.243 points. Exclusive breastfeeding consists of giving only breast milk to a baby without adding water or other liquids such as teas, sugar water, and fruit juice or other foods before 6 months after birth. Only medications are allowed. Immediate exclusive breastfeeding has a positive and powerful effect on the probability of child survival. This result is consistent with those found by other authors [23–25].

Regarding mixed feeding, the mother gives her breast milk and complements with bottle feeding (infant formula) to feed her child. The coefficient associated with this variable is positive and very significant (α_2_ = 0.4388, *P* < 0.01). All things being equal when the mother associates the best milk to supplement the feeding, the child’s chances of survival increase by 0.07 points.

The coefficient associated with the variable “breastfeeding with other liquids” is positive and not significant.

After controlling socio-economic characteristics of mothers, the results contained in Table 3 suggest several comments:

Firstly, the coefficients associated with “Immediate exclusive breastfeeding” remains always positive and very significant (α_1_ = 0.1663, *P* < 0.01). Exclusive breastfeeding provides a much greater chance of survival for the child [18]. Likewise, the coefficients associated with variable “mixed feeding” is positive and very significant (α_2_ = 0.4645, *P* < 0.01). “Breastfeeding and other liquid” (α_3_ = 0.0832, *P* > 0.1) still are not significant.

Secondly, the coefficients associated with variables “vitamins” (α_4_ = 0.0093, *P* > 0.1) and “alcohol” (α_5_ = 0.0893, *P* > 0.1) are not significant. In Côte d’Ivoire, vitamin A supplements are given to children from 0 to 5 years old by health workers preventively. Thus, 6 children from 6 to 59 months old over 10 received supplements of vitamin A [26]. This result justifies that the semi-solid food that mothers give to their infants is low in vitamins.

Thirdly, the probability of survival increases when a mother has at least a primary school education. Indeed, all things being equal, when a mother attended primary school (β_1_ = 0.0809, *P* < 0.15) or secondary and higher (β_2_ = 0.1842, *P* < 0.05), her child is more likely to survive. The secondary and higher level of education has a strong positive effect than the primary school level. Indeed, the probability of survival increases from 0.012 points and 0.024 points respectively. Formal education is a determining factor in the decrease in child mortality [15].

Fourthly, the status of women in the household significantly influences the survival of a child. All things being equal, when the mother lives with a spouse (γ_1_ = 0.0736, *P* < 0.15); it means that the husband is the head of the household, the probability of child survival increases by 0.011 points. When she plays the same role (γ_2_ = 0.2344, *P* < 0.05), the chance of the child’s survival increase by 0.023 points, all things being equal (see Table 3). Whether a mother lives with a spouse or not, her child is more likely to survive. Indeed, the mother remains the leading actor in caring for children in terms of caring for illness, monitoring, and supervision. In other words, she remains the children’s key worker and contributes to the improvement of their condition, source of fulfillment, physical, and psychological growth of child [27].

Lastly, the child’s chances of survival increase with a healthy environment (δ = 0.1373, *P* < 0.01). Other things being equal, the probability of survival increases by 0.02 points when the child’s place of residence is very healthy. The living environment is the environment in which the child lives and grows with the best conditions including hygiene [15]. The variable “no access to drinking water” has no effect on child’s survival chances (η = − 0.0052, *P* > 0.1).

## Discussion

### The effect of type of breastfeeding on the probability of child survival

The econometric results show that immediate exclusive breastfeeding has a positive and strong effect on the probability of child survival after controlling socio-economic determinants. According to WHO [2], exclusive breastfeeding is one of the prevention methods for reducing child mortality. Breastfeeding is also a means of prevention of delayed growth. Despite the benefits of breastfeeding, it is hard to believe that less than half of newborns in the world benefit from it and much less are those who are exclusively breastfed in the first 6 months of life [1]. At the global level, only 38% of infants are exclusively breastfed during the first 6 months [1]. In Côte d’Ivoire, the statistics show that 97% of infants are breastfed. However, only 12% of children under 6 months old are exclusively breastfed and 64% of children whose age is between 6 and 9 months received complementary food and 21% did not receive supplementary feeding [26]. Exclusive breastfeeding stops at 6 months after birth, after this period, breastfeeding should continue until the age of two at least and be associated with complementary food rich in vitamin, protein, iron, etc.

When a mother associates breast milk with baby milk bottles, the chances of child survival can increase.

It is practiced mainly in two situations: either the bottle comes in complement of the breast if the mother does not have enough breast milk or she is busy with economic activities. A time constraint leads the women to associate breast milk and artificial milk to feed their babies.

### The relevant socio-economic determinants of the probability of child survival

The study highlights the socio-economic factors that influence the child’s survival chances. Higher level of mother’s education is a major determinant of child survival [28]. The positive effect of formal education on the probability of child survival can be explained in two ways: firstly, women with at least a basic level of education, who can read and write, choose good formula milk to complement the breastfeeding. This result is consistent with previous studies that reveal that maternal schooling is positively associated with good feeding varieties of children [29–31]. Women who have acquired a high level of education adopt good dietary practices in healthy hygienic conditions. They choose proper combinations of foods to bring more calories to their respective infants [32].

Secondly, formal education is a better factor for women’s economic empowerment. Indeed, a high level of education allows women to easily get access to the labor market with high employability [33]. In other words, the probability of gainful employment is very high. As a result, with wage employment, she becomes financially independent and put her baby in good conditions of living. Empirical researches indicate that when a woman has financial power, child nutrition, health, and education improve [34–36]. Woman’s socio-economic status contributes effectively to the nutritional status of children. In the same way, the first place occupied by a woman in the household has more a positive and significant effect on the probability of child survival compared to a role played by a man [37].

Furthermore, a healthy environment has a positive and very significant impact on the child’s survival chances. A healthy environment takes into account the type of habitat, hygiene within the housing, access to clean and modern toilets, and healthy eating practices. A child who develops in such an environment is more likely to be uninfected by microbes, bacteria and viruses, and agents of disease transmission [32].

### Limitations

The conduct of this study comes up against limitations because of a certain lack of information contained in the database.

Firstly, among the independent variables, monetary income as an indicator of well-being could have been used to classify households by quartile or quintile of poverty. However, Demographic and Health Surveys do not collect information on household expenditures or incomes. As a result, consumption per capita cannot be taken as an indicator of standard of living. Income remains the best indicator of well-being [38]. We did not take into account this variable in the model.

Likewise, DHS does not collect information on food prices that may affect the allocation of household resources. This information is very difficult to obtain in existing databases in Côte d’Ivoire. As a result, we did not consider the price of food commodities among the explanatory variables.

Secondly, the important role played by the mother in the nutrition of children aged from 0 to 5 years old could have been more explicit if the key variables on women’s autonomy in all its dimensions (decision-making, financial autonomy, freedom to move, etc.) would be taken into account in the model [29, 35]. Fortunately, we have considered the variable “woman is a head of household” which captures a little autonomy of woman.

## Conclusion

The study highlights the key role that breastfeeding plays in the survival of children with ages between 0 and 5 years old. Formal education, a child’s care by his/her mother, and a healthy environment are the main socio-economic determinants of child survival. In addition, a child’s body needs micronutrients contained in breast milk to function better and provide an immune defense. However, from 6 months, one must complete infants’ feeding by providing some best milk formula rich in vitamins, proteins, minerals, etc.

Modernism influences young mothers especially in urban areas in the practice of breastfeeding and also the customs and traditions influence the rural areas in terms of exclusive breastfeeding. Thus, we must strengthen programs to promote exclusive breastfeeding up to 6 months after birth by social campaigns through the media. It should also strengthen the capacity of health workers (midwives, nurses, doctors, etc.) to better guide and provide training to mothers and young women of childbearing age to allow them to practice exclusive breastfeeding immediately. It is also important to educate women so as to improve hygiene in their housing, in their neighborhood, and in their community in order to promote the well-being and health of their children.

## Additional file

**Additional file1 1.** Probit regression results and marginal effects after probit.

## Data Availability

The datasets analyzed during the current study available from the corresponding author on reasonable request. I inform you that I obtained the database by requesting for DHS data of Côte d’Ivoire to DHS archive program (archive@dhsprogram.com).

## Abbreviations

DHS CI-III: Third Demographic and Health Survey in Côte d’Ivoire
DHS: Demographic Health Survey
HIV/AIDS: Human immunodeficiency virus/acquired immune deficiency syndrome
INS: National Institute of Statistics
MDGs: Millennium Development Goals
MICS: Multiple Indicator Cluster Survey
STIs: Sexually transmitted infections
UNICEF: United Nations International Children’s Emergency Fund
WHO: World Health Organization

## Footnotes

1 The news fell at the Council of Ministers on Wednesday 15 February 2012. The Ivorian authorities have just made free delivery and caesarean section for all women of Côte d’Ivoire. On the presentation of the Minister of Health and the fight against AIDS, the President of the Republic signed a decree exempting the payment of benefits and acts in connection with low birth and cesarean delivery in public health establishments [5].

2 The presentation is inspired by Lachaud [14].
