# Supplementary material for: Breastfeeding and child survival from 0 to 5 years in Côte d'Ivoire
Source: J Health Popul Nutr. 2020 Mar 30;39:5. doi: 10.1186/s41043-020-0210-4 (PMC7106562; doi:10.1186/s41043-020-0210-4)
Supplement: Supplementary file 1 [file 41043_2020_210_MOESM1_ESM.docx]

```
. probit B5  allait1 mixte alaitem

Iteration 0:   log likelihood = -2313.3482
Iteration 1:   log likelihood = -2225.1444
Iteration 2:   log likelihood = -2224.1248
Iteration 3:   log likelihood = -2224.1245
Iteration 4:   log likelihood = -2224.1245

Probit regression                                 Number of obs   =       7776
                                                  LR chi2(3)      =     178.45
                                                  Prob > chi2     =     0.0000
Log likelihood = -2224.1245                       Pseudo R2       =     0.0386
```

B5	Coef.	Std. Err.	z	P>|z|	[95% Conf.	Interval]
allait1	.1740916	.0501836	3.47	0.001	.0757335	.2724497
mixte	.438842	.0766446	5.73	0.000	.2886213	.5890627
alaitem	.1012365	.0774571	1.31	0.191	-.0505767	.2530496
_cons	1.062981	.0292225	36.38	0.000	1.005706	1.120256

```
. mfx compute

Marginal effects after probit
      y  = Pr(B5) (predict)
         =  .91993564
```

variable	dy/dx	Std. Err.	z	P>|z|	[95% C.I.	]	X
allait1*	.0243619	.00658	3.70	0.000	.011462	.037262	.248328
mixte*	.0682174	.01242	5.49	0.000	.043871	.092564	.560442
alaitem*	.0151052	.01159	1.30	0.193	-.007615	.037826	.51839

(*) dy/dx is for discrete change of dummy variable from 0 to 1

```
. probit B5 allait1 alaitmixte alaitem educ2 educ3 femconj femchef  alcool vitam
> ines habitat eausec

Iteration 0:   log likelihood = -2313.3482
Iteration 1:   log likelihood = -2210.0124
Iteration 2:   log likelihood = -2208.5262
Iteration 3:   log likelihood = -2208.5253
Iteration 4:   log likelihood = -2208.5253

Probit regression                                 Number of obs   =       7776
                                                  LR chi2(11)     =     209.65
                                                  Prob > chi2     =     0.0000
Log likelihood = -2208.5253                       Pseudo R2       =     0.0453
```

B5	Coef.	Std. Err.	z	P>|z|	[95% Conf.	Interval]
allait1	.1663109	.0505184	3.29	0.001	.0672965	.2653252
alaitmixte	.4644881	.0818936	5.67	0.000	.3039797	.6249966
alaitem	.0831662	.0873284	0.95	0.341	-.0879943	.2543268
educ2	.0809435	.0513705	1.58	0.115	-.0197407	.1816277
educ3	.1842388	.0802949	2.29	0.022	.0268638	.3416139
femconj	.0736057	.0491519	1.50	0.134	-.0227303	.1699417
femchef	.2344112	.1058211	2.22	0.027	.0270056	.4418168
alcool	.0892893	.128554	0.69	0.487	-.1626719	.3412505
vitamines	.009297	.0854507	0.11	0.913	-.1581833	.1767773
habitat	.1373305	.0392412	3.50	0.000	.0604192	.2142418
eausec	-.0051728	.0082399	-0.63	0.530	-.0213227	.0109771
_cons	.7351766	.0864696	8.50	0.000	.5656993	.9046539

```
. mfx compute

Marginal effects after probit
      y  = Pr(B5) (predict)
         =  .92130729
```

variable	dy/dx	Std. Err.	z	P>|z|	[95%	C.I.]	X
allait1*	.0230247	.00657	3.50	0.000	.010143	.035906	.248328
alaitm~e*	.0715102	.0132	5.42	0.000	.045642	.097378	.560442
alaitem*	.0122407	.01289	0.95	0.342	-.013016	.037498	.51839
educ2*	.0115233	.00709	1.63	0.104	-.002364	.02541	.229938
educ3*	.0243061	.00944	2.57	0.010	.005805	.042807	.093493
femconj*	.0110621	.00756	1.46	0.143	-.003746	.02587	.72338
femchef*	.0296059	.01132	2.62	0.009	.007427	.051785	.053241
alcool*	.0123634	.01674	0.74	0.460	-.020454	.045181	.03948
vitami~s*	.0013578	.01241	0.11	0.913	-.022974	.02569	.099794
habitat	.0201634	.00575	3.51	0.000	.008897	.031429	1.79038
eausec	-.0007595	.00121	-0.63	0.530	-.00313	.001611	3.45589

(*) dy/dx is for discrete change of dummy variable from 0 to 1

```
. probit B5 allait1 alaitmixte alaitem educ2 educ3 femconj femchef  alcool vitam
> ines habitat eausec nvie1 nvie2 nvie3 nvie4

Iteration 0:   log likelihood = -2313.3482
Iteration 1:   log likelihood = -2209.6955
Iteration 2:   log likelihood = -2208.1969
Iteration 3:   log likelihood = -2208.1959
Iteration 4:   log likelihood = -2208.1959

Probit regression                                 Number of obs   =       7776
                                                  LR chi2(15)     =     210.30
                                                  Prob > chi2     =     0.0000
Log likelihood = -2208.1959                       Pseudo R2       =     0.0455
```

B5	Coef.	Std. Err.	z	P>|z|	[95% Conf.	Interval]
allait1	.1660034	.0505821	3.28	0.001	.0668644	.2651424
alaitmixte	.4648908	.0818999	5.68	0.000	.30437	.6254117
alaitem	.0811662	.087405	0.93	0.353	-.0901444	.2524768
educ2	.0841864	.0515952	1.63	0.103	-.0169383	.1853111
educ3	.1904374	.0819199	2.32	0.020	.0298773	.3509975
femconj	.0726558	.0493049	1.47	0.141	-.0239801	.1692917
femchef	.2319544	.1060017	2.19	0.029	.0241949	.439714
alcool	.0925398	.1289078	0.72	0.473	-.1601147	.3451944
vitamines	.0119871	.0859066	0.14	0.889	-.1563867	.180361
habitat	.1431615	.0473	3.03	0.002	.0504553	.2358677
eausec	-.0052085	.0082559	-0.63	0.528	-.0213898	.0109729
nvie1	.019684	.0873045	0.23	0.822	-.1514296	.1907976
nvie2	.0156018	.0779265	0.20	0.841	-.1371313	.1683349
nvie3	.0505858	.0785085	0.64	0.519	-.1032881	.2044596
nvie4	.0065191	.0787532	0.08	0.934	-.1478345	.1608726
_cons	.7039662	.1324263	5.32	0.000	.4444154	.963517
